# Egg Protein Transferrin-Derived Peptides IRW and IQW Regulate *Citrobacter rodentium*-Induced, Inflammation-Related Microbial and Metabolomic Profiles

**DOI:** 10.3389/fmicb.2019.00643

**Published:** 2019-04-03

**Authors:** Yong Ma, Sujuan Ding, Gang Liu, Jun Fang, Wenxin Yan, Veeramuthu Duraipandiyan, Naif Abdullah Al-Dhabi, Galal Ali Esmail, Hongmei Jiang

**Affiliations:** ^1^College of Bioscience and Biotechnology, Hunan Agricultural University, Changsha, China; ^2^Hunan Provincial Key Laboratory of Animal Nutritional Physiology and Metabolic Process, CAS Key Laboratory of Agro-ecological Processes in Subtropical Region, National Engineering Laboratory for Pollution Control and Waste Utilization in Livestock and Poultry Production, Institute of Subtropical Agriculture, Chinese Academy of Sciences, Changsha, China; ^3^Department of Botany and Microbiology, College of Sciences, King Saud University, Riyadh, Saudi Arabia

**Keywords:** IRW, IQW, *Citrobacter rodentium*, inflammation, microbial, metabolomic

## Abstract

Bioactive peptides that target the gastrointestinal tract can strongly affect the health of animals and humans. This study aimed to evaluate the abilities of two peptides derived from egg albumin transferrin, IRW and IQW, to treat enteritis in a mouse model of *Citrobacter rodentium*-induced colitis by evaluating serum metabolomics and gut microbes. Forty-eight mice were randomly assigned to six groups: basal diet (CTRL), intragastric administration *Citrobacter rodentium* (CR), basal diet with 0.03%IRW (IRW), CR with 0.03% IRW (IRW+CR), basal diet with 0.03%IQW (IQW) and CR with 0.03% IQW (IQW+CR). CR administration began on day 10 and continued for 7 days. After 14 days of IRW and IQW treatment, serum was collected and subjected to a metabolomics analysis. The length and weight of each colon were measured, and the colon contents were collected for 16srRNA sequencing. The colons were significantly longer in the CR group, compared to the CTRL group. A serum metabolomics analysis revealed no significant difference in microbial diversity between the six groups. Compared with the CTRL group, the proportions of Firmicutes and Actinobacteria species decreased significantly and the proportions of Bacteroidetes and Proteobacteria species increased in the CR group. There were no significant differences between the CTRL and other groups. The serum metabolomics analysis revealed that Infected by *CR* increased the levels of oxalic acid, homogentisic acid and prostaglandin but decreased the levels of L-glutamine, L-acetyl carnitine, 1-methylhistidine and gentisic acid. Therefore, treatment with IRW and IQW was shown to regulate the intestinal microorganisms associated with colonic inflammation and serum metabolite levels, thus improving intestinal health.

## Introduction

*Citrobacter rodentium* (CR) is a natural mouse pathogen that can be used to cause intestinal inflammation replaced enteropathogenic *Escherichia coli* (EHEC) and enteropathogenic *E. coli* (EPEC) (Hart et al., 2008). Although CR is a nonmotile pathogen, it can infect the host by passing through the intestinal mucosa to reach the intestinal epithelial cells. It adheres to the intestinal epithelial cells through surface structures called fimbriae when the CR started to infect the host (Mundy et al., 2003; Hart et al., 2008). These fimbriae can mediate infections in target cells that result in disorders of the host immune system. Therefore, the fimbriae are widely considered an important pathogenic factor in several diseases, particularly urinary, genital, and gastrointestinal infections (Petty et al., 2010). CR successfully colonizes intestinal epithelial cells, where it causes significant increases in the levels of cytokines and infiltrating immune cells in the intestinal environment (Collins et al., 2014). Consequently, infected intestinal epithelial cells promote the apical expression of antimicrobial peptides and inducible nitric oxide synthase, thereby modulating the inflammatory signal to produce NO, which inhibits CR (Vallance et al., 2002; Lopez et al., 2016). Although CR induces inflammatory infiltration, proinflammatory factor production, intestinal mucosal injury, and similar processes associated with inflammatory bowel disease in mice, this pathogen does not cause clinical diarrhea in humans (MacDonald et al., 2003). It may be an ideal model for mice but may not translate to human colonic inflammation, as it does not cause diarrhea in man (Zhang W. et al., 2015).

Many studies have provided substantial evidence indicating the regulatory roles of active peptides in intestinal inflammation (Kvidera et al., 2017; Eissa et al., 2018; Lv et al., 2018). The results of mouse experiments showed that porcine β-defensin 2 might improve mucosal lesions and extracellular permeability by targeting the NF-κB pathway and could thus alleviate inflammatory enteritis (Han et al., 2015). Cathelicidin-related antimicrobial peptide (CRAMP) restore body weight loss caused by ulcerative colitis and maintain colonic epithelial integrity (Li et al., 2018). Cathelicidin-BF can effectively inhibit the phosphorylation of NF-κB and increase the intestinal barrier function to reduce inflammation (Zhang H. et al., 2015). Egg protein transferrin-derived peptides, such as IRW and IQW, have been shown to provide effective relief of cardiovascular disease symptoms. The intact tripeptide structure of these molecules can alleviate endothelial inflammation and oxidative stress (Chen et al., 2017). IRW and IQW have also been shown to alleviate inflammatory responses induced by tumor necrosis factor (TNF) and may therefore be useful as nutraceuticals (Majumder et al., 2013). IRW can inhibit the TNF-α-induced expression of both ICAM-1 and VCAM-1, whereas IQW can only inhibit the expression of ICAM-1. Treatment with IRW and IQW also inhibits the expression of antioxidant enzymes and promotes the activity of superoxide dismutase (SOD), catalase (CAT), and glutathione peroxidase (GPx) (Liu et al., 2018; Ma et al., 2018). Therefore, we hypothesized that IRW and IQW could reduce intestinal damage and thus slow the inflammatory process. This study mainly analyzed the levels of serum metabolites in a *CR*-induced model of IBD in response to these two peptides, as well as the effects on microbial diversity and composition in the colonic contents.

## Materials and Methods

### Animal and Experimental Design

The Animal Care and Use Committees of Hunan Agricultural University provided approval for the experiments. IRW and IQW (Ontores, Zhejiang, China) were dissolved in sterile water at 4°C until the solution temperature reached equilibrium with the ambient temperature. Both peptides were synthetic and had purity levels of 99%. A CR bacterial solution was mixed with glycerol at a ratio of 3:7 and stored at -80°C before inoculation into Luria Bertani broth to generate an activation culture before gavage. The mature CR culture was centrifuged at 4000 rpm for 10 min, and the supernatant was mixed with physiological saline to yield a 5 × 10^9^ CFU/ml bacterial suspension that was set aside (Guan et al., 2016). Forty-eight mice (average body weight: 23 g; age: 8 weeks) were randomly assigned to six groups (*n* = 8 per group): basal diet (CTRL), basal diet with intragastric administration of 0.1 mL bacterial suspension (CR), basal diet with 0.03%IRW (IRW), CR with 0.03% IRW (IRW+CR), basal diet with 0.03%IQW (IQW) and CR with 0.03% IQW (IQW+CR) (Liu et al., 2018). All mice in one group were housed and fed in seperate cages. The cages were stored in a sterile environment under a 12-h light-dark cycle, relative humidity of 53%, and temperature of 24°C. After 3 days of acclimation, IRW and IQW were fed to mice. CR administration began on day 10 and continued for 7 days. All mice were slaughtered on day 17. The experimental process adhered strictly to the animal experimental guidelines. Serum samples were collected for metabolomics analysis. The length and weight of the colon were measured, and a section was collected for a morphological analysis. The colon contents were also collected for 16s rDNA sequencing.

### Histopathology

Colon fragments were rinsed with saline and fixed in 12% formalin. The fixed colon fragments were dehydrated using an ethanol gradient and embedded in paraffin. Ten-micrometer sections were stained with hematoxylin and eosin according to standard protocols. The samples were observed under blind conditions using an optic Olympus BX41 microscope (Münster, Germany).

### Serum Metabolomics

The serum samples were thawed at room temperature and 100-μL aliquots were transferred into centrifuge tubes (1.5 mL). All serum samples were extracted using 300 μL of methanol and mixed with 10 μL of an internal standard (3.1 mg/mL, DL-o-chlorophenylalanine). The samples were then vortexed for 30 s and centrifuged at 12000 rpm and 4°C for 15 min. ACQUITY^TM^ UPLC -QTOF analysis system and Waters ACQUITY UPLC HSS T3 chromatographic column (2.1 mm × 100 mm, 1.8 covering m) were used for LC-MS detection. The following chromatographic separation conditions were applied: column temperature, 40°C; mobile phase A, water+0.1% formic acid; mobile phase B: acetonitrile+0.1% formic acid; flow rate: 0.35 mL/min; injection volume: 6 μL. The data were first transformed to CDF files using CDF bridge and imported into XCMS software for peak picking, peak alignment, peak filtering and peak filling. The data, including the retention time (RT), MZ, observations (samples) and peak intensity, were normalized using Excel 2007.

### 16S rDNA and Illumina MiSeq Sequencing

The colon contents were collected from all mice immediately after sacrifice. The V3–V4 region of the 16sRNA gene was sequenced in the 48 content samples. Microbial DNA was extracted from the colon contents using the QIAamp DNA Stool Mini Kit (Qiagen, Hilden, Germany). The microbial V3-V4 region was amplified by PCR using the primers 5′-ACTCCTACGGGAGGCAGCA and 3′-GGACT ACHVGGGTWTCTAAT. Amplicons were extracted from a 2% agarose gel using the TIANgel MIdi Purification Kit (TIANGEN BIOTECH, Beijing, China) and were subsequently purified. For detailed experimental steps on Illumina MiSeq sequencing, please see our previous article (Zhu et al., 2018); The general data analysis was performed by a commercial company (Novogene, Beijing, China).

### Correlation Analysis Between Differential Metabolite Levels and Genus-Level Intestinal Microbes

SPSS 16.0 was used to conduct a correlative analysis of the proportions of microorganisms in each genus and details of the metabolic differences among all samples. GraphPad Prism was used to conduct a regression analysis of the results from the correlation analysis and draw graphs.

### Data Analysis

All data are presented as means ± standard errors of the means (SEM) and were analyzed using SPSS 16.0 software. Differences between mean values were evaluated using a one-way analysis of variance. If dissimilarities were detected, Tukey’s multiple comparisons test was used. A *P* value <0.05 was considered to indicate a significant difference.

## Results

### Effects of IRW and IQW on Changes in the Colon

**Figure 1:**
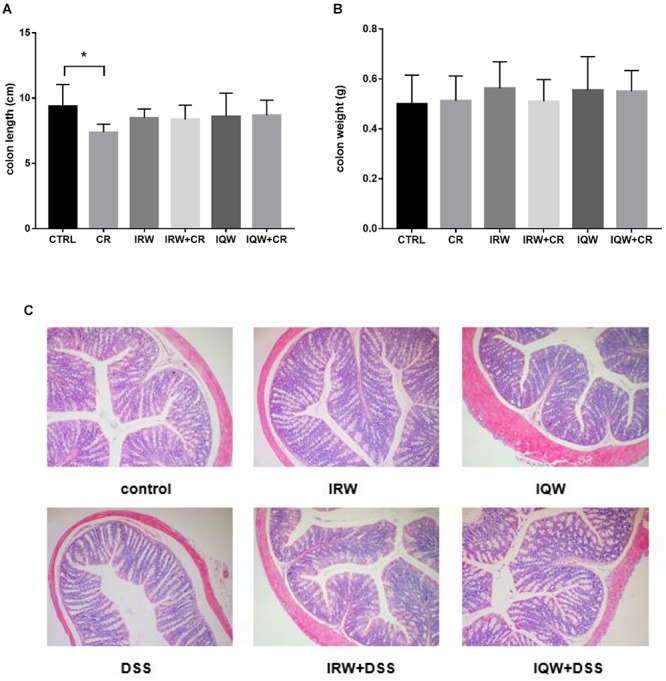
Effects of IRW and IQW on **(A)** colon length and **(B)** colon weight. **(C)** Representative photomicrographs of colon sections stained with hematoxylin and eosin (×100 magnification). Data are shown as means ± standard errors of the means. *N* = 8, ^∗^*P* < 0.05.

Infected by CR induced ulcers and other inflammatory reactions that caused mucosal damage in the colon and reduced the colon length. The average colon length in the CTRL group was 9.36 ± 1.68 cm, compared to 7.38 ± 0.61 cm in the CR group. In other words, CR treatment significantly reduced the colon length (*P* < 0.05), whereas co-treatment with IRW and IQW maintained the colon length (*P* > 0.05) (Figure 1A). However, the colon weights did not differ between the six groups (*P* > 0.05) (Figure 1B). More severe histological damage was observed in colon tissues from the CR group, compared with the CTRL group, as demonstrated by broad disruption of the tissue architecture and the disappearance of intestinal crypts and goblet cells. Compared with the CTRL group, colon tissue from the CR group exhibited more extensive cellular infiltration (Figure 1C).

### Diversity of Bacterial Community of Colonic Contents

**Figure 2:**
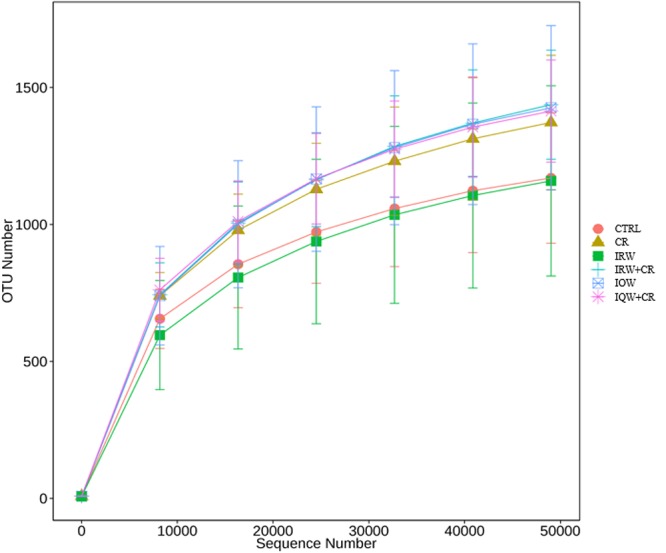
Pan-species analysis curve corresponding to the total OTU number and sample size.

Table 1 presents the original readings obtained by sequencing the V3–V4 region of 16s rRNA from each sample of colon contents. The sample size we sequenced is sufficient to reflect the abundance of microorganisms. The OTU (Operational Taxonomic Units) number of microbes in the colon is about 1200 (Figure 2). Raw reads and Clean Reads in the CR group were lower than other groups (Table 1). The six groups of goods coverage have reached more than 99%. There were no statistically significant differences in community richness (chao1 and ACE), community diversity (Shannon and Simpson) or species coverage (cargo coverage) (Table 1) (*P* > 0.05).

**Table 1:** Alpha diversity indices of fecal bacterial communities of mice.

	CTRL	CR	IRW	IRW+CR	IQW	IQW+CR
Raw_reads	85186.75 ± 2517.01	82072.88 ± 4917.86	85372.38 ± 1894.51	88655.25 ± 5995.91	84431.88 ± 5436.38	89921.50 ± 8418.42
Clean_Reads	80204.37 ± 117.48	78722.63 ± ± 4523.63	80060.75 ± 268.08	81782.25 ± 5147.64	80263.00 ± 3899.80	83648.50 ± 7008.54
Observed species	1169.9 ± 254.4167	1372.5 ± 261.9406	1426.0 ± 320.7420	1413.9 ± 199.1872	1159.1 ± 371.2440	1437.4 ± 212.9144
Goods coverage	0.9951 ± 0.0016	0.9935 ± 0.0032	0.9935 ± 0.0009	0.9938 ± 0.0012	0.9943 ± 0.0014	0.9929 ± 0.0016
Shannon	6.6685 ± 0.5191	6.8589 ± 0.4850	6.5415 ± 1.3152	6.9744 ± 0.5201	5.8018 ± 1.3015	6.8660 ± 0.52770
Simpson	0.9630 ± 0.0132	0.9608 ± 0.0198	0.9244 ± 0.1000	0.9685 ± 0.0101	0.9054 ± 0.0756	0.9699 ± 0.0093
Chao1	1324.5 ± 302.4212	1788.7 ± 892.4182	1612.2 ± 329.4143	1610.1 ± 213.6898	1343.5 ± 387.572	1697.8 ± 230.7201
ACE	1357.3 ± 302.5543	1649.6 ± 443.7628	1668.9 ± 325.3484	1649.3 ± 207.4422	1395.4 ± 391.6893	1735.7 ± 250.6430

### Composition of Bacterial Communities in the Colon Contents

**Figure 3:**
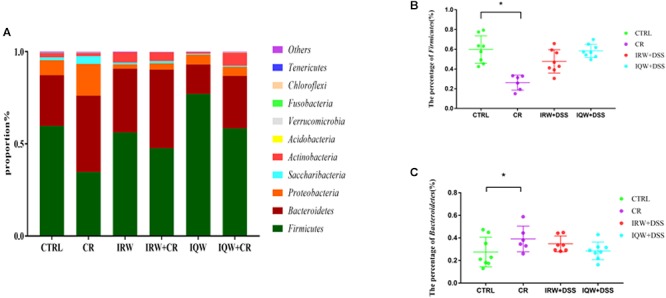
**(A)** Taxonomic compositions of fecal bacterial communities at the phylum level. **(B)** Percentage of Firmicutes species in a sample from each of the four groups. **(C)** Percentage of Bacteroidetes species in a sample from each the four groups. ^∗^*P* < 0.05.

**Figure 4:**
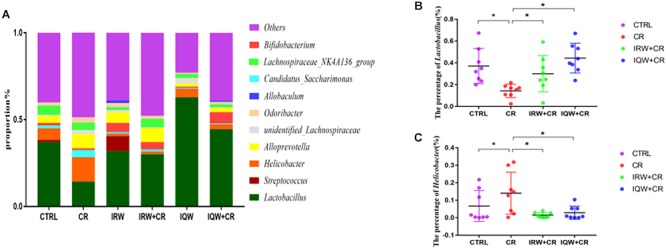
**(A)** Taxonomic compositions of the fecal bacterial communities at the genus level. **(B)** Percentage of *Lactobacillus* species in a sample from each of the four groups. **(C)** Percentage of *Helicobacter* species in a sample from each of the four groups. ^∗^*P* < 0.05.

We performed a taxonomic analysis of the sequencing results to determine the composition and community structure of different populations (or samples) at each classification level (e.g., domain, kingdom, gate, class, sequence, family, genus, species, OTU). At the phylum level, 10 phyla were detected at the highest abundance. Firmicutes, Bacteroidetes, Proteobacteria, Saccharibacteria and Actinobacteria were the most abundantly expressed phyla in all groups, accounting for 99% of all sequencing results. In the CTRL group, Firmicutes (59.7%), Bacteroidetes (27.5%) and Proteobacteria (8.2%) were the most abundant phyla (Figure 3), whereas Firmicutes (34.8%), Bacteroidetes (41.4%), Proteobacteria (17.2%) and Saccharibacteria (4.2%) were the most abundant phyla in the CR group (Figure 3). Firmicutes (56.1%), Bacteroidetes (34.6%), Proteobacteria (2.3%) and Actinobacteria (5.6%) were the most abundant phyla in the IRW group (Figure 3), while Firmicutes (47.8%), Bacteroidetes (42.6%), Proteobacteria (3.4%) and Actinobacteria (4.7%) were the most abundant phyla in the IRW+CR group (Figure 3). Firmicutes (77.1%), Bacteroidetes (15.9%) and Proteobacteria (5.0%) were the most abundant phyla in the IQW group (Figure 3), while Firmicutes (58.4%), Bacteroidetes (28.5%) and Proteobacteria (5.0%) were the most abundant in the IQW+CR group (Figure 3). Compared with the CTRL group, the CR group exhibited significantly lower percentages of Firmicutes (59.7% vs. 34.8%) and Actinobacteria species (2.3% vs. 1.7%) (Figure 3) but significantly higher percentages of Bacteroidetes (27.5% vs. 41.4%), Proteobacteria (8.2% vs. 17.2%) and Saccharibacteria species (1.5% vs. 4.2%) (Figure 3). However, treatment with IRW and IQW restored the percentages of Firmicutes, Actinobacteria, Bacteroidetes, Proteobacteria and Saccharibacteria in CR animals to levels that did not differ significantly from the CTRL group (Figure 3). Figure 4 lists the 10 most abundant genera in the colonic contents. Notably, the percentage of *Lactobacillus* decreased and the percentages of *Helicobacter* and *Odoribacter* increased in the CR group, compared with the CTRL group (Figure 4). Similarly, IRW and IQW treatment restored the normal percentages of *Lactobacillus*, *Helicobacter* and *Odoribacter* species in CR animals.

### Analysis of Serum Metabolomics

**Figure 5:**
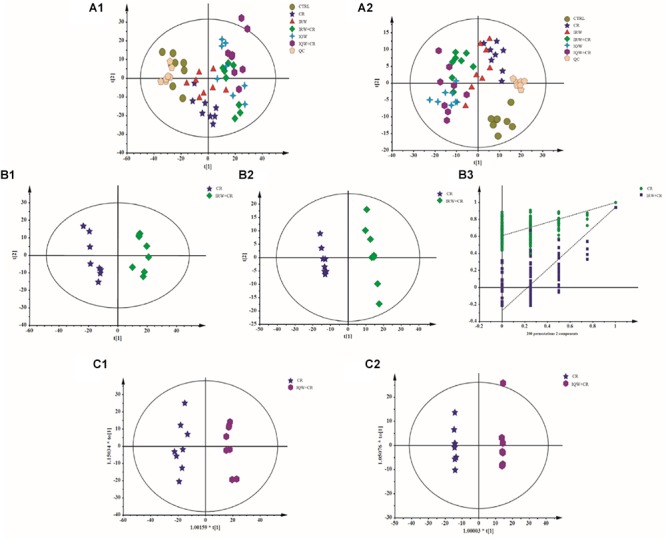
Plots of the multivariate statistical comparisons between groups. **(A1)** A PCA score plot of all samples (ESI+). **(A2)** PCA score plot of all samples (ESI-). **(B1)** PLS-DA score plot of CR-IRW+CR (ESI+). **(B2)** PLS-DA score plot of CR-IRW+CR (ESI-). **(B3)** PLS-DA score plot of CR-IRW+CR. **(C1)** OPLS-DA score plot of CR-IQW+CR (ESI+). **(C2)** OPLS-DA score plot of CR-IQW+CR (ESI-).

To identify differences between the six groups, a PCA (Principal Component Analysis) modeling method was used to analyze the serum samples. In this analysis, six principal components were obtained in the positive mode, and the performance characteristics of this multivariate model in the positive ion mode were as follows: R^2^X = 0.705, *Q*^2^ = 0.591. Nine principal components were obtained in the negative mode. Similarly, the performance characteristics of the model in the negative ion mode were as follows: R^2^X = 0.531, *Q*^2^ = 0.189. In addition, the total PCA score demonstrates that the scatters of the six groups were completely separated in both the ESI+ (Figure 5A1) and ESI- modes (Figure 5A2). There were 10 metabolic differences between the CR and CTRL groups, including four in the ESI+ mode and six in the ESI- mode (Table 2). The results of a plasma metabolite analysis revealed decreased levels of oxalic acid, allantoin, homogentisic acid, α-linolenic Acid, prostaglandin D3 and lysoPC and increased amounts of L-glutamine, gentisic acid, L-acetylcarnitine and 1-methylhistidine in the CR group. However, normal amounts of L-glutamine, gentisic acid, L-acetylcarnitine and 1-methylhistidine were observed in the IRW+CR and IQW+CR groups (Table 2).

**Table 2:** Metabolomic changes in the plasma in the CTRL, CR, IRW+CR and IQW+CR.

			CTRL *VS* CR			CR *VS* IRW+CR			CR *VS* IQW+CR		
	mz	RT (min)	VIP	*P*-value	Change	VIP	*P*-value	Change	VIP	*P*-value	Change
Oxalic acid	91.0112	0.803	1.2256	0.00358	↓	1.5708	0.0002423	↓	1.2652	0.002392	↓
Allantoin	159.0608	0.813	1.1943	0.004939	↓	–	–	–	1.2441	0.003026	↓
Homogentisic acid	169.0458	1.353	1.4419	0.000181	↓	–	–	–	–	–	–
α-Linolenic Acid	301.2143	12.543	1.6374	0.000001	↓	–	–	–	–	–	–
L-Glutamine	148.0711	0.869	1.2717	0.016882	↑	1.7659	0.000003	↓	1.5234	0.000036	↓
Gentisic acid	188.9878	4.162	1.0928	0.046417	↑	–	–	–	–	–	–
L-Acetylcarnitine	202.0892	6.043	1.3159	0.012622	↑	1.5666	0.000259	↓	–	–	–
1-Methylhistidine	204.0556	6.592	1.3608	0.009214	↑	1.5500	0.000333	↓	–	–	–
Caprylic acid	167.1098	2.949	–	–	–	1.7543	0.000005	↓	1.7982	0.000001	↓
Pregnenolone	317.2443	17.736	–	–	–	1.2245	0.010607	↑	1.5992	0.000004	↑
Prostaglandin D3	385.1666	12.535	2.0430	0	↓	–	–	–	–	–	–
L-Tryptophan	227.0855	0.8	1.2311	0.003374	↓	–	–	–	–	–	–
LysoPC(10:0)	447.2007	18.016	1.2534	0.018938	↓	–	–	–	–	–	–

When the CR group received IRW treatment, we further adopted a supervised multidimensional statistical method, namely the partial least square discriminant analysis (PLS-DA), to conduct statistical analysis of the two groups of samples. The model quality parameters were two principal components in the positive mode, R^2^X = 0.449, R^2^Y = 0.967, *Q*^2^ = 0.904, and two principal components in the negative mode, R^2^X = 0.34, R^2^Y = 0.998, *Q*^2^ = 0.942. The PLS-DA loading plot demonstrates the complete separation of the scatters of the two groups (Figure 5B1,B2) due to differences in the detected metabolites. When the CR group received IQW treatment, the OPLS-DA method was again used for the modeling analysis. One principal component and one orthogonal component, R^2^X = 0.481, R^2^Y = 0.979, *Q*^2^ = 0.918, were obtained in the positive mode. One principal component and two orthogonal components, R^2^X = 0.472, R^2^Y = 1, *Q*^2^ = 0.941, were obtained in the negative mode. The OPLS-DA scoring diagrams are shown in Figure 5C1,C2. In order to verify whether the model is “overfitting”, the model is sorted (Figure 5B3), and the results show that the model is reliable.

### Correlation Analysis Between Differential Metabolites and Genus-Level Intestinal Microbes

**Figure 6:**
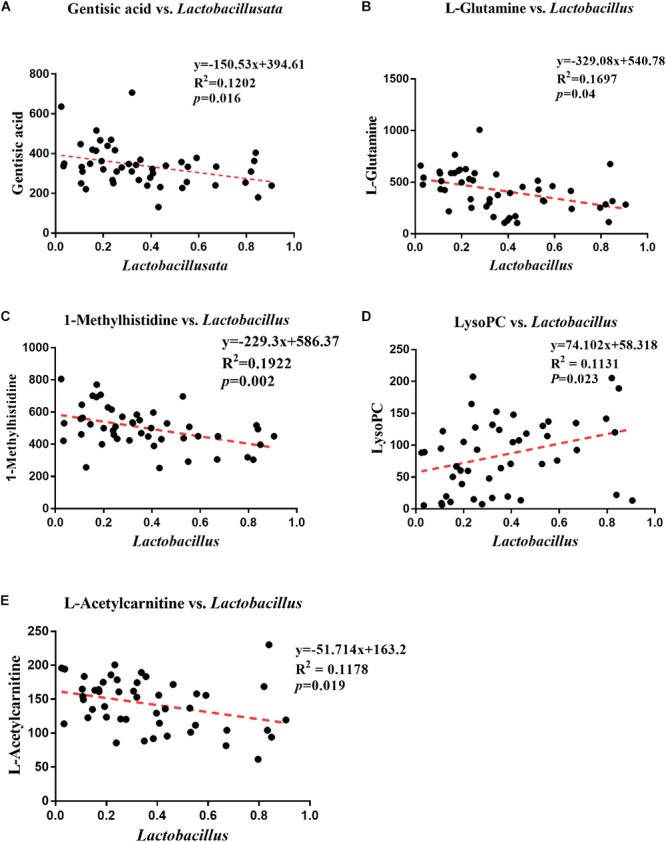
Analysis of correlations between differential metabolites and genus-level intestinal microbes. **(A)** Correlation analysis between *Lactobacillus* and gentisic acid. **(B)** Correlation analysis between *Lactobacillus* and L-glutamine. **(C)** Correlation analysis between *Lactobacillus* and 1-methylhistidine. **(D)** Correlation analysis between *Lactobacillus* and LysoPC. **(E)** Correlation analysis between *Lactobacillus* and L-acetylcarnitine.

The correlations between different metabolites in the serum metabolome results and the profiles of genus-level intestinal microbes. Gentisic acid, L-glutamine, 1-methylhistidine, lysoPC and L-acetylcarnitine were found to associate significantly with *Lactobacillus*. Specifically, negative correlations were detected between *Lactobacillus* and gentisic acid (Pearson correlation: -0.347, *P* = 0.016) (Figure 6A), L-glutamine (Pearson correlation: -0.412, *P* = 0.004) (Figure 6B), 1-methylhistidine (Pearson Correlation: -0.438, *P* = 0.002) (Figure 6C), L-acetylcarnitine (Pearson correlation: -0.338, *P* = 0.019) (Figure 6E). However, a positive correlation was observed between *Lactobacillus* and LysoPC (Pearson correlation: 0.328, *P* = 0.023) (Figure 6D).

## Discussion

*Citrobacter rodentium* causes severe inflammatory infections in the mouse colon (Guan et al., 2016). These infections cause mucosal damage, reduce the colonic length, increased the colonic wall thickness, and cause hyperemia and even ulceration (Liu et al., 2015). Some studies also suggest that shortening of the colon is closely related to histological changes and even suggest that this morphological parameter indicates the degree of colonic inflammation (Yuge et al., 2014; Oz, 2017a). In our study, the colon lengths of mice infected intragastrically with CR were significantly shorter than those of normal mice, and this change was eliminated in the presence of IRW and IQW. The shortening of colon length may be an early warning of inflammation. A large number of experimental results indicate that colonic inflammation is accompanied by a decrease in colon length and other different degrees of morphological damage (Gearry et al., 2009; Gadaleta et al., 2011). Accordingly, IRW and IQW treatment appears to play a role in colonic inflammation (Jiao et al., 2019).

Mouse fecal microbes may be contaminated, so we try to be aseptic as possible during the sampling process and the samples are stored in sterile, enzyme-free EP tubes. Our microbiological sequencing results are consistent with the composition of the gut microbiota in most inflammatory studies, so the possibility of contamination is low. The intestinal microbes change significantly at the time of intestinal inflammation. However, different degrees of inflammation lead to different results in dissimilar individuals; for example, IBD ecological disorders differ widely (Walker et al., 2011; Weichselbaum and Klein, 2018). Notably, colonic inflammation commonly results in decreases in microbial diversity and the proportion of Firmicutes, as well as an increase in the proportion of Proteobacteria (Matsuoka and Kanai, 2015; Hong and Piao, 2018). In this study, the lack of significant changes in microbial diversity may be attributable to large differences in diversity within the group, a consequence of poor homogeneity among the mice. Fortunately, the proportion of Firmicutes was lower and the proportion of Proteobacteria was higher in the CR group, compared to the CTRL group. CR played a role in establishing a model of colonic inflammation. The proportions of Firmicutes and Proteobacteria returned to normal, however, in mice treated with IRW and IQW, which demonstrates the usefulness of these treatments. *Lactobacillus* and *Bifidobacterium* are the most commonly used probiotics (Pace et al., 2015; Azad et al., 2018b). Some studies have shown that *Lactobacillus* degrades pro-inflammatory factors secreted in milk proteins, thus eliminating inflammation (von Schillde et al., 2012; Yin et al., 2018). Furthermore, a study by Hormannsperger et al. (2013) found that *Lactobacillus* not only degraded pro-inflammatory chemokines and reduced immune cell infiltration in colonic inflammation (Di Cerbo and Palmieri, 2013; Lightfoot et al., 2015) but could also be used to identify protective microbial structures and even in new drug applications (Hormannsperger et al., 2013; Li and Gui, 2018; Zhang and Gui, 2018). This study also confirmed that the proportions of *Lactobacillus* and *Bifidobacterium* were significantly reduced in the CR group but returned to normal after treatment with IRW and IQW. *Helicobacter* spp. cause more severe intestinal inflammation and injury via M1 macrophages (Krakowiak et al., 2015; Azad et al., 2018a; Ding et al., 2019). Other studies have shown that *Helicobacter* can improve ROS levels by inducing regeneration synthesis and neutrophil infiltration of the epithelium both *in vivo* and *in vitro* (Davies et al., 1994; Bagchi et al., 1996; Mannick et al., 1996). The proportion of inflammatory *Helicobacter* species was significantly increased in the CR group, whereas this increase was inhibited by IRW and IQW. Accordingly, these peptides improved the intestinal condition.

When serum metabolomics were measured in normal and colitis mice, significant differences were observed (Schicho et al., 2010). Some studies have found that inflammatory infections can alter the structure of the gut microbiota and regulate the corresponding serum amino acids accordingly. As the proportion of *Helicobacter* increased, the concentration of tryptophan decreased, consistent with the changing trends in our study results. This finding suggests that *Helicobacter* can inhibit tryptophan metabolism (Gadaleta et al., 2011). Catabolic enzymes and indoleamine 2,3-dioxygenase (IDO) can metabolize tryptophan to kynurenine (Munn et al., 1998; Puccetti and Grohmann, 2007), and tryptophan metabolism was shown to affect immune modulation in recent studies (Romani et al., 2008; Fu et al., 2018). The tryptophan concentration may be inversely proportional to the disease activity and CRP level; in other words, a decrease in tryptophan is associated with inflammation (Hisamatsu et al., 2012). Hashimoto et al. found that caprylic acid could inhibit transcription of the gene encoding IL-8 in Caco-2 cells (Hoshimoto et al., 2002), thereby inhibiting the chemotaxis and proliferation of inflammatory cells. This phenomenon was also found to increase mitochondrial respiration in inflammatory cells (Hecker et al., 2014; Oz, 2017b). The expression of caprylic acid improved significantly after IRW and IQW were administered to CR mice, indicating that these protein transferrin-derived peptides play a role in CR-induced intestinal inflammation. Specifically, these peptides alter the composition of gut microbes that regulate metabolites. In turn, these metabolites increase the level of caprylic acid and thus improve the symptoms of inflammation. *Lactobacillus* can have good antibacterial activity against *Clostridium* (Monteiro et al., 2019), and can well reduce the oxidative stress caused by high-fat diet (Hsu et al., 2019). *Lactobacillus* has been shown to produce SCFA (short chain fatty acid) from L-glutamine (Botta et al., 2017). In humans, SCFA can regulate immune and metabolic functions in multiple tissues and organs (Ohira et al., 2017; Bin et al., 2018). Therefore, we believe that the amount of L-glutamine decreases (due to increased consumption) as the number of *Lactobacillus* increases, leading to a negative correlation between these parameters. The correlation between these species and metabolites indicates that the strain can promote the production of some inhibitory substances or can use these substances to produce metabolites to inhibit inflammation, thereby alleviating the inflammation of the colon.

## Conclusion

In conclusion, supplementation of IRW and IQW improves the morphological damage of the colon caused by CR, while regulating the microbial composition in the colon and altering the serum metabolites through changes in microbial composition. Finally, colonic inflammation is improved by changes in these metabolite components. Further studies are needed to clarify and confirm the benefits of IRW and IQW treatment in the host gastrointestinal tract and other organ systems.

## Author Contributions

YM and SD carried out the study and performed the statistical analysis. VD, NA-D, and GE provided assistance for the experiments. GL, JF, and HJ designed the research. YM, SD, and WY prepared the first draft of the manuscript. GL and JF read and revised the manuscript.

## Conflict of Interest Statement

The authors declare that the research was conducted in the absence of any commercial or financial relationships that could be construed as a potential conflict of interest.
